# Comparison of Dynamic Measures in Intraoperative Goal-Directed Fluid Therapy of Patients with Morbid Obesity Undergoing Laparoscopic Sleeve Gastrectomy

**DOI:** 10.1007/s11695-024-07154-z

**Published:** 2024-03-21

**Authors:** Gökhan Urhan, İsmail Demirel, Ahmet Deniz, Ahmet Aksu, Aysun Yıldız Altun, Esef Bolat, Azize Beştaş, Gülsüm Altuntaş

**Affiliations:** 1 Anesthesiology and Reanimation Department, Elazığ Fethi Sekin City Hospital, Elazig, Turkey; 2 Anesthesiology and Reanimation Department, School of Medicine, Firat University, Elazig, 23119 Turkey; 3 Anesthesiology and Reanimation Department, Medicine Faculty, Firat University, Elazig, Turkey

**Keywords:** Morbid obesity, Goal-directed fluid therapy, Laparoscopic sleeve gastrectomy, PVI, PPV, SPV, Lactate

## Abstract

**Introduction:**

Obesity increases the risk of morbidity and mortality during surgical procedures. Goal-directed fluid therapy (GDFT) is a new concept for perioperative fluid management that has been shown to improve patient prognosis. This study aimed to investigate the role of the Pleth Variability Index (PVI), systolic pressure variation (SPV), and pulse pressure variation (PPV) in maintaining tissue perfusion and renal function during GDFT management in patients undergoing laparoscopic sleeve gastrectomy (LSG).

**Materials and Methods:**

Two hundred ten patients were enrolled in our prospective randomized controlled clinical trial. Demographic data, hemodynamic parameters, biochemical parameters, the amount of crystalloid and colloid fluid administered intraoperatively, and the technique of goal-directed fluid management used were recorded. Patients were randomly divided into three groups: PVI (*n* = 70), PPV (*n* = 70), and SPV (*n* = 70), according to the technique of goal-directed fluid management. Postoperative nausea and vomiting, time of return of bowel movement, and hospital stay duration were recorded.

**Results:**

There was no statistically significant difference between the number of crystalloids administered in all three groups. However, the amount of colloid administered was statistically significantly lower in the SPV group than in the PVI group, and there was no significant difference in the other groups. Statistically, there was no significant difference between the groups in plasma lactate, blood urea, and creatinine levels.

**Conclusion:**

In LSG, dynamic measurement techniques such as PVI, SPV, and PPV can be used in patients with morbid obesity without causing intraoperative and postoperative complications. PVI may be preferred over other invasive methods because it is noninvasive.

**Graphical abstract:**

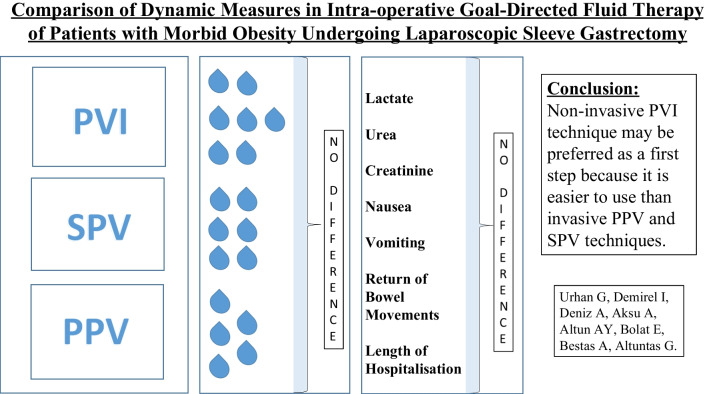

## Introduction

The goal of perioperative fluid management should be to ensure intravascular volume balance and continuity, tissue perfusion, adequate O_2_ supply, normal electrolyte concentrations, and normoglycemia. For this purpose, crystalloid and/or colloid fluids may be administered intravenously.

In recent years, goal-directed fluid therapy (GDFT) management has come to the forefront to avoid hypervolemia in the intraoperative period and to ensure patient optimization in the postoperative period [1]. GDFT can be performed by monitoring intravascular volume with hemodynamic measurement techniques such as plethysmographic waveform and arterial pressure wave analysis [2]. Although dynamic measurements based on the cardiopulmonary relationship have been reported to be ideal techniques for fluid therapy management in patients receiving mechanical ventilation, it has also been reported that dynamic measurement techniques may not be sufficient to predict fluid requirements in cases where there is a decrease in pulmonary compliance [3].

In the intraoperative period, excessive and uncontrolled fluid infusion can lead to adverse conditions such as hypertension, pulmonary edema, cerebral edema, peripheral edema, periorbital edema, bowel dysfunction, and delayed wound healing. Inadequate fluid intake can lead to decreased cardiac output and impaired tissue perfusion, shock, and multiorgan failure [4, 5]. GDFT protocols in surgical patients have been reported to prevent intraoperative fluid overload and insufficiency and reduce postoperative complications [6].

Obesity is associated with increased total and lean body mass; however, intracellular, extracellular, and absolute total body fluids tend to be relatively reduced in individuals with obesity compared with those with normal weight. Data regarding the most effective fluid management for patients with morbid obesity are insufficient. Some studies indicate the potential harm to patients due to excessive fluid loading in traditional practices; nevertheless, there is a limited number of studies investigating GDFT in the context of bariatric surgery [7].

It is well known that GDFT administers less fluid in the intraoperative period compared with standard fluid therapy strategies. Accordingly, the incidence of surgical site infections is reduced, and the resumption of bowel movements is accelerated [8]. It has been reported that the use of dynamic parameters instead of static parameters, such as mean arterial pressure (MAP), central venous pressure (CVP), and urine monitoring in intraoperative fluid management in patients undergoing surgical procedures, leads to a reduction in perioperative complications and a shortening of hospital stay [9, 10].

In the Pleth Variability Index (PVI) technique, one of the dynamic parameters used to evaluate intravascular volume status, respiratory changes are measured using plethysmography. Another dynamic parameter, systolic pressure variation (SPV), is the difference between the maximum systolic arterial pressure (SAPmax) and the minimum systolic arterial pressure (SAPmin) in a respiratory cycle. Finally, pulse pressure variation (PPV), one of the dynamic parameters, is calculated by measuring the highest (PPmax) and the lowest value (PPmin) of pulse pressure in a respiratory cycle.

Although many studies in the literature compare GDFT management with standard fluid management, studies comparing different dynamic measurement techniques, both invasive and noninvasive, used in the follow-up of GDFT management, especially in bariatric surgery, are very limited. Accordingly, our study aimed to investigate the advantages of PVI, SPV, and PPV techniques, the preferred dynamic measurement techniques for managing GDFT in patients undergoing laparoscopic sleeve gastrectomy (LSG) in the postoperative period.

## Material-Method

The study was approved by the Firat University Non-Interventional Research Ethics Committee (dated 19/12/2018, number 300693). Between February 1, 2019, and August 1, 2019, a total of 210 patients with morbid obesity, who provided written consent, were prospectively enrolled in our randomized controlled clinical study at the Department of General Surgery, Firat University Hospital.

Those who had undergone bariatric surgery other than sleeve gastrectomy, were aged younger than 18 years, had cardiac arrhythmias, had a cardiac ejection fraction < 30%, had pulmonary pathology that did not allow tidal volume formation greater than 6 mL/kg, and had hepatic and renal dysfunction and patients with American Society of Anesthesiologist (ASA) physical status ASA-3 were excluded from the study.

The 210 patients who were enrolled in the study were randomly divided into three groups using the sealed envelope method: PVI (*n* = 70), PPV (*n* = 70), and SPV (*n* = 70), depending on which measurement technique was used during the follow-up of the GDFT.

In our study, PVI measurements were performed using Masimo Set version V7.1.1.5 pulse oximetry (MasimoCo, Irvine, California) to continuously, automatically, and noninvasively measure plethysmographic changes during the respiratory cycle. PPV and SPV were measured continuously and automatically using a CARESCAPE B650 model monitor (General Electric Healthcare, USA) using arterial pressure wave analysis via a 20-G radial artery cannula.

Regardless of the groups, 2–3 mg/kg propofol (propofol 1%, Fresenius, Germany) corresponding to total body weight (TBW), 1 µg/kg remifentanil (Ultiva®, GSK, USA) corresponding to ideal body weight (IBW), and 0.6–0.8 mg/kg rocuronium bromide (Esmeron®, MSD, Germany) were used during the induction of general anesthesia. Endotracheal intubation was performed after an adequate depth of anesthesia was achieved. During maintenance anesthesia, 0.05–0.2 µg/kg remifentanil infusion and 2–2.5% sevoflurane (Sevorane®, liquid 100%, Abbvie, England) were administered with a 50:50% medical air: O_2_ mixture according to the IBW. Rocuronium bromide was used as a muscle relaxant for maintenance if needed. In this standard general anesthesia protocol, the patient was ventilated with an end-tidal CO_2_, between 35 and 45 mmHg, a tidal volume of 8 mL/kg according to the IBW, and an I:E (inspiration-to-expiration) ratio of 1:2 to maintain normocapnia. At the end of the surgery, the residual effect of the intraoperatively administered muscle relaxant was eliminated using 2 mg/kg sugammadex (Bridion®, MSD, Netherlands), according to the intravenous IBW.

The same surgical team performed the surgery on all patients in the study, and the intra-abdominal pressure was maintained below 15 mmHg.

Patients in the PVI group (*n* = 70) received 500 mL of crystalloid during induction and a 2 mL/kg/h crystalloid infusion corresponding to the TBW. If the PVI exceeded 13% for more than 5 min, 250 mL bolus colloid infusions were administered until the PVI was 13%, and the colloid doses were recorded. Patients whose mean arterial pressure could not be brought to 65 mmHg despite a PVI value of 13% or less were administered norepinephrine.

Patients in the SPV group (*n* = 70) received 500 mL of crystalloid during induction and a crystalloid infusion of 2 mL/kg/h, according to TBW. If the SPV was above 10 mmHg for more than 5 min, 250 mL bolus colloid infusions were administered until the SPV was 10 mmHg, and the colloid doses were recorded. Patients whose SPV value decreased to 10 mmHg or less but whose mean arterial pressure value of 65 mmHg could not be achieved were administered norepinephrine.

Patients in the PPV group (*n* = 70) received 500 mL crystalloid during induction and a crystalloid infusion of 2 mL/kg/h corresponding to TBW. When PPV exceeded 13% for more than 5 min, 250 mL bolus colloid infusions were administered until PPV was 13%, and the colloid doses were recorded. Patients whose mean arterial pressure could not be reduced to 65 mmHg despite a PPV value of 13% or less were administered norepinephrine.

In our study, lactate concentrations were recorded and compared in blood samples taken from patients during skin incision, at the beginning of each hour during surgery, and at 6, 12, and 24 h postoperatively to assess tissue perfusion. We used 0.9% NaCl as a crystalloid and succinylated gelatin (Gelofusine®, B. Braun) as a colloid. Blood urea and creatinine levels were recorded preoperatively and postoperatively at week 24 to assess renal function. Postoperative nausea and vomiting score, time of return of bowel movements, tracking of bowel sounds, time of first gas leakage, and number of days of hospital stay were recorded.

In line with the primary objective of our study, a sample size analysis was conducted using the G*power software (Version 3.1) to determine the number of patients required for inclusion in the research. The sample size determination analysis drew upon literature information and expert opinions. As a result of the conducted sample size analysis, a decision was made to include a total of 210 patients in the sample, considering an 80% power (1-β = 0.80) and α = 0.05 error value (95% confidence interval).

## Statistical Analysis

All analyses were performed using the IBM SPSS Statistics Version 22.0 statistical software package. Categorical variables are expressed as numbers and percentages, whereas continuous variables are summarized as mean and standard deviation and median and minimum–maximum where appropriate. The chi-squared test was used to compare categorical variables between the groups. The normality of distribution for continuous variables was confirmed using the Kolmogorov–Smirnov test. For comparison of more than two groups, one-way analysis of variance (ANOVA) or the Kruskal–Wallis test was used depending on whether the statistical hypotheses were fulfilled. For normally distributed data, regarding the homogeneity of variances, the Bonferroni, Scheffe, and Tamhane tests were used for multiple comparisons of groups. For non-normally distributed data, the Bonferroni-adjusted Mann–Whitney *U* test was used for multiple comparisons of groups. To evaluate the change in the measurements obtained in the time interval, repeated measurement analysis was applied. The statistical level of significance for all tests was considered as 0.05.

## Results

**Fig. 1:**
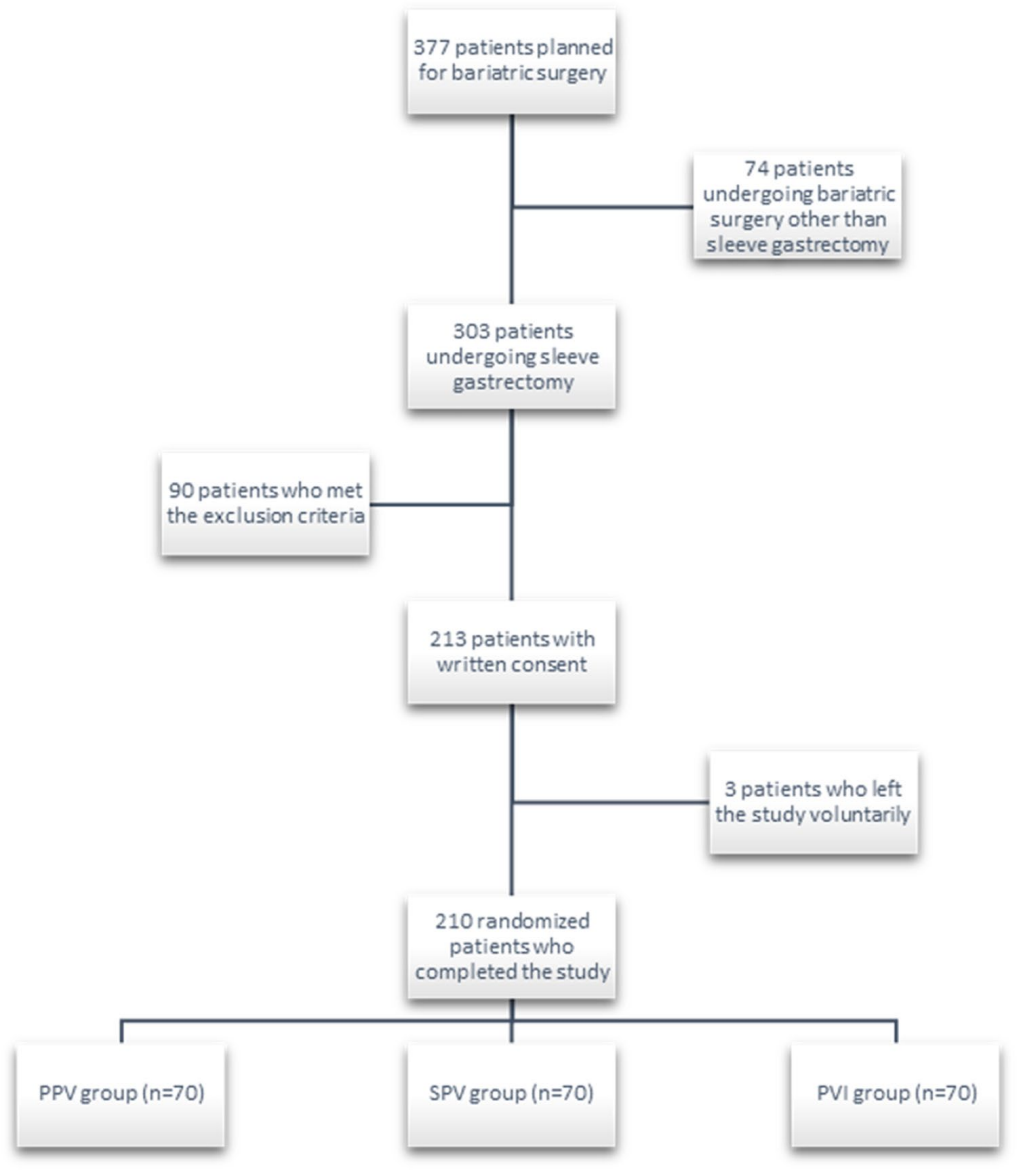
Study participation process

The flow of patients’ participation in the study is shown in Fig. 1.

When comparing the patients’ demographic data (age, height, weight, body mass index (BMI), sex), no significant difference was found between the groups (Table 1).

**Table 1:** Demographic data

	Group			*p* value
	PVI (*n* = 70)	SPV (*n* = 70)	PPV (*n* = 70)	
Age	35.04 ± 10.065	35.06 ± 11.544	36.99 ± 10.927	0.470
Weight	120.69 ± 22.321	121.61 ± 26.790	117.40 ± 17.437	0.501
Height	165.83 ± 9.731	167.36 ± 9.523	165.73 ± 8.672	0.512
BMI	43.731 ± 6.313	43.7479 ± 6.166	42.7286 ± 5.533	0.514
Sex (female/male)	54/16	48/22	54/16	0.408

When comparing the amount of crystalloid administered to the patients during the intraoperative period, there was no statistically significant difference between the groups (*p* = 0.598). However, when comparing the amount of colloid administered intraoperatively, it was found that the mean amount was statistically significantly higher in the PVI group than in the SPV group (*p* = 0.010*). No other pairwise comparisons showed a significant difference (Table 2).

**Table 2:** Amount of fluid given to patients intraoperatively

	Group			*p* value
	PVI (*n* = 70)	SPV (*n* = 70)	PPV (*n* = 70)	
Crystalloid (mL)	790.91 ± 80.94	804.57 ± 97.34	793.30 ± 75.15	0.598
Colloid (mL)	364.28 ± 239.58	239.28 ± 234.81	303.57 ± 247.79	0.010*

^*^Between PVI and SPV

**Fig. 2:**
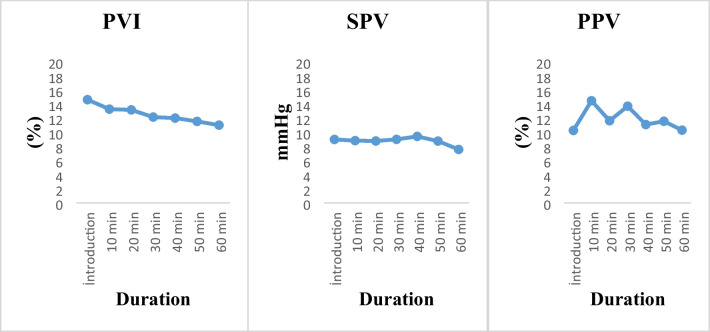
PVI, SPV, and PPV analyses in the intraoperative period

The intraoperative PVI, SPV, and PPV values are shown in Fig. 2.

No statistically significant difference was found when intraoperative and postoperative lactate values were compared between the groups (*p* = 0.250) (Table 3). The decrease in lactate levels over time in the postoperative period was statistically significant in all groups (*p* < 0.001) (Table 3).

**Table 3:** Lactate values (mmol/L)

		Group			In-group time change (*p* value)
		PVI (*n* = 70)	SPV (*n* = 70)	PPV (*n* = 70)	
Intraoperative	Introduction	1.393 ± 0.511	1.197 ± 0.436	1341 ± 0.641	< 0.001*
	1st hour	1.490 ± 0.525	1.297 ± 0.402	1.293 ± 0.490	
Postoperative	6th hour	2.559 ± 1.032	2.547 ± 0.859	2.573 ± 1.052	
	12th hour	2.264 ± 0.887	2.053 ± 0.632	2.250 ± 0.887	
	24th hour	1.549 ± 0.705	1.484 ± 0.493	1.564 ± 0.489	
Between the groups (*p* value)		0.250			

* Means statistically significant

No statistically significant difference was found when comparing urea and creatinine levels between groups (*p* = 0.369 and *p* = 0.771, respectively) (Table 4). In addition, it was found that preoperative urea and creatinine levels were statistically significantly higher than urea and creatinine levels at the 24th postoperative hour in all groups (*p* = 0.001).

**Table 4:** Urea-creatinine values of the patient groups

		Group			*p* value for time change
		PVI (*n* = 70)	SPV (*n* = 70)	PPV (*n* = 70)	
Urea (mg/dL)	Preoperative	32.56 ± 20.83	29.09 ± 10.43	28.44 ± 9.49	< 0.001*
	Postoperative	17.04 ± 5.63	18.56 ± 855	16.61 ± 11.03	
	*p* value for the group	0.369			
Creatinine (mg/dL)	Preoperative	0.672 ± 0.136	0.698 ± 0.150	0.698 ± 0.179	< 0.001*
	Postoperative	0.644 ± 0.137	0.650 ± 0.151	0.650 ± 0.211	
	*p* value for the group	0.771			

* Means statistically significant

It was found that there was no statistically significant difference in postoperative nausea and vomiting scores, return of bowel movements, and hospital stay of patients (Tables 5 and 6).

**Table 5:** Nausea-vomiting in patient groups

		Group			Total	*p* value
		PVI (*n* = 70)	SPV (*n* = 70)	PPV (*n* = 70)		
Nausea	None	10 (14.3%)	14 (20.0%)	10 (14.3%)	34 (16.2%)	0.888
	0–2 h	11 (15.7%)	7 (10.0%)	8 (11.4%)	26 (12.4%)	
	2–6 h	24 (34.3%)	23 (32.9%)	27 (38.6%)	74 (35.2%)	
	6–24 h	25 (35.7%)	26 (37.1%)	25 (35.7%)	76 (36.2%)	
Vomiting	None	41 (58.6%)	43 (61.4%)	38 (54.3%)	122 (58.51%)	0.956
	0–2 h	2 (2.9%)	2 (2.9%)	3 (4.3%)	7 (3.3%)	
	2–6 h	6 (8.6%)	6 (8.6%)	9 (12.9%)	21 (10.0%)	
	6–24 h	21 (30.0%)	19 (27.1%)	20 (28.6%)	60 (28.6%)

**Table 6:** Return of bowel movements and length of hospitalization in patient groups

	Group			*p* value
	PVI (*n* = 70)	SPV (*n* = 70)	PPV (*n* = 70)	
	Median (min–max)	Median (min–max)	Median (min–max)	
Gas (days)	2 (1–4)	2 (1–3)	2 (1–3)	0.119
Stool (days)	4 (2–5)	4 (2–5)	4 (1–5)	0.587
Length of hospitalization (days)	6 (4–10)	7 (5–9)	7 (4–16)	0.186

## Discussion

Studies have attracted attention in recent years showing that perioperative fluid therapy management directly affects patient prognosis. GDFT has been shown to reduce postoperative complications and shorten hospital stays [11]. Therefore, various fluid therapy management protocols are used [12]. The protocols used in GDFT have been reported to reduce patient morbidity and mortality [13]; however, there are few protocols in the guidelines for fluid management in bariatric surgery.

If fluid therapy is inadequate, complications such as hypotension, acute renal injury, arrhythmia, ischemia, and anastomotic leakage may occur due to hypovolemia. In contrast, complications such as prolonged mechanical ventilation, delayed wound healing, infection, and prolonged discharge time due to hypervolemia may occur after excessive fluid intake [14, 15].

Nowadays, bariatric surgical procedures are mostly performed with laparoscopic techniques, and it is well known that hemodynamic disturbances may occur during such procedures due to insufflation and the patient’s position. However, it has been reported that these deteriorations are not evident in the clinic when the pressure is 15 mmHg or less [16]. As long as the intra-abdominal pressure does not exceed 15 mmHg during laparoscopic procedures, the decrease in cardiac output is minimal and does not affect the clinical outcome in healthy subjects [17]. As in these studies, surgical procedures in our study were performed with insufflation pressures of 15 mmHg and below.

Recent studies found no difference between the choice of crystalloids or colloids in GDFT strategies regarding complications, morbidity, and mortality [18, 19]. Moreover, although it has been shown that the choice of crystalloid and colloid solutions does not cause a significant difference in mortality rates, it has also been found that the choice of hydroxyethyl starch (HES) as a colloid may increase mortality [20]. Accordingly, we used 0.9% NaCl as a crystalloid and succinylated gelatin (Gelofusine®, B. Braun) as a colloid in our study. Although the amount of colloid used was statistically lower in the SPV group in the present study, we do think it was not clinically significant.

It has been reported that PVI is safe in assessing fluid responsiveness in perioperative patient groups. However, the noninvasive technique of PVI used in GDFT is distinct from invasive techniques. Nevertheless, it is suggested that noninvasive techniques such as PVI may be inadequate in patient groups with vascular tone and volume changes [21, 22].

Studies have reported that SPV and PPV-controlled fluid management, invasive GDFT techniques, successfully predict response to fluid therapy [23–26]. In another study, PPV, goal-directed therapy, and standard fluid therapy were compared regarding postoperative complications and hospital stay. It was found that there was no difference in complication rates, and the length of hospital stay decreased in the GDFT group [23].

Despite these studies in the literature, there are few studies on GDFT management in patients with morbid obesity. Although the dynamic parameters differed in our study, a similar protocol was used in the patients. Our study used a threshold of 13% for PVI-directed fluid therapy, 10 mmHg for SPV-directed fluid therapy, and 13% for PPV-directed fluid therapy under the thresholds used in the literature [11, 27].

There is a significant relationship between intravascular volume insufficiency, tissue hypoxia, and lactate levels. Measurement of plasma lactate levels, one of the methods used to assess tissue perfusion, is a sensitive technique, even though it is indirect [28]. The reason why the measurement of lactate levels is preferred in evaluating the efficiency of fluid resuscitation is the normalization of plasma lactate levels as a result of meeting fluid requirements and providing adequate cardiac preload [29]. We used plasma lactate values to evaluate tissue perfusion and blood urea and creatinine values to evaluate renal functions.

## Conclusion

As a result, it was found that there was no superiority among the three groups in terms of tissue perfusion and renal function. In light of these findings, it was determined that adequate intravascular volume was provided, and renal perfusion was preserved in all three groups. For LSG, it was found that there was no difference between the goal-directed fluid therapies using PVI, SPV, and PPV measurement techniques in terms of lactate levels, urea-creatinine levels, return time of bowel movements, and hospital stay. In this case, the noninvasive PVI measurement technique may be preferred as a first step because it is easier to use than the invasive PPV and SPV measurement techniques. Although GDFT protocols are known to prevent intraoperative fluid overload in bariatric surgery, further research is needed to optimize dynamic measurement techniques that automatically and continuously display variable results.

## Data Availability

All data was reported in the manuscript.
